# Cebidae *Alu* Element Alignments and a Complex Non-Human Primate Radiation

**DOI:** 10.3390/life12101655

**Published:** 2022-10-20

**Authors:** Jessica M. Storer, Jerilyn A. Walker, Morgan A. Brown, Mark A. Batzer

**Affiliations:** 1 Department of Biological Sciences, Louisiana State University, 202 Life Sciences Building, Baton Rouge, LA 70803, USA; 2 Institute for Systems Biology, Seattle, WA 98109, USA

**Keywords:** Cebidae, *Alu*, phylogeny, platyrrhine

## Abstract

**Simple Summary:**

Relationships among the small monkeys living in the Americas such as the squirrel monkeys, owl monkeys (the ‘night monkeys’), marmosets and capuchins (the ‘organ grinder’ monkeys) are still under debate. This study utilized multi-species DNA sequence alignments to investigate these relationships. Each alignment contained a unique *Alu* element insertion, a genetic marker considered ‘identical by descent’ such that the absence of the *Alu* element is the ancestral state. *Alu* element diversity reveals that the emergence and radiation of these different primate lineages was rapid and complex. The close relationship between squirrel monkey and capuchins was confirmed while the placement of owl monkey with respect to the marmoset remains unresolved. The results of this study will assist in research and conservation strategies for America’s monkeys.

**Abstract:**

Phylogenetic relationships among Cebidae species of platyrrhine primates are presently under debate. Studies prior to whole genome sequence (WGS) availability utilizing unidirectional *Alu* repeats linked *Callithrix* and *Saguinus* as sister taxa, based on a limited number of genetic markers and specimens, while the relative positions of *Cebus*, *Saimiri* and *Aotus* remained controversial. Multiple WGS allowed computational detection of *Alu*-genome junctions, however random mutation and evolutionary decay of these short-read segments prevented phylogenetic resolution. In this study, WGS for four Cebidae genomes of marmoset, squirrel monkey, owl monkey and capuchin were analyzed for full-length *Alu* elements and each locus was compared to the other three genomes in all possible combinations using orthologous region sequence alignments. Over 2000 candidates were aligned and subjected to visual inspection. Approximately 34% passed inspection and were considered shared in their respective category, 48% failed due to the target being present in all four genomes, having N’s in the sequence or other sequence quality anomalies, and 18% were determined to represent near parallel insertions (NP). Wet bench locus specific PCR confirmed the presence of shared *Alu* insertions in all phylogenetically informative categories, providing evidence of extensive incomplete lineage sorting (ILS) and an abundance of *Alu* proliferation during the complex radiation of Cebidae taxa.

## 1. Introduction

While the relationships among the Platyrrhini families Cebidae, Atelidae, and Pitheciidae are supported as monophyletic, the relationship among the genera of Cebidae remains controversial. Previous analyses of platyrrhine phylogeny using *Alu* insertions as phylogenetic markers, although informative, were limited in their scope due to the lack of assembled genomes at the time [1,2,3,4]. Other investigations have attempted to resolve Cebidae phylogeny, particularly the placement of *Aotus*, using a few *Alu* insertions [2,5] or other methods [6], and none have resulted in complete agreement. Additional studies have highlighted the problematic position of *Aotus* within Cebidae and have explored different techniques such as Restriction-site Associated DNA sequencing (RAD-seq) to find maximum likelihood relationships and have still been unsuccessful at confidently positioning *Aotus* [7]. This inconsistency has been attributed to near simultaneous branching events that occurred 19–20 million years ago (mya) leading to the rapid radiation of cebid species over a short evolutionary time of 1–2 million years (my) [8]. The difficult phylogenetic placement of *Aotus* has also been attributed to a large ancestral effective population size, rather than concurrent speciation events [9]. Various proposed phylogenies among cebid taxa are illustrated in [5,8,9] and reviewed in detail in [8].

*Alu* element detection methods based on *Alu*-genome junctions, such as the polyDetect program [10] have been applied to the Cebidae lineage of platyrrhine phylogeny [11]. However, the short reads did not allow for sufficient homology to accurately predict shared *Alu* insertions across genera diverged by ~20 my [3,12]. To overcome the impact of evolutionary random genomic decay across lineages, the current study uses the full-length *Alu* sequences extracted from the assembled genomes along with 600 bp of flanking unique DNA sequence. The genomes of the same four cebid species used in Storer et al. (2020) [11] (marmoset, squirrel monkey, capuchin monkey and owl monkey), were used to computationally ascertain all possible *Alu* insertions from the available genome assemblies and align them to the other three genomes. Full-length *Alu* sequence and a combined total of 1200 bp of flanking sequence provides adequate homology for accurate multiple sequence alignments. Another advantage to using full-length *Alu* sequence, compared to short read data, is that it allows for better refinement of *Alu* subfamilies to provide a clearer indication of shared *Alu* insertions across an evolutionary time scale based on *Alu* age.

## 2. Materials and Methods

### 2.1. Lineage-Specific Alu Elements

Each of four platyrrhine genomes (common marmoset; [caljac3], capuchin monkey; [Cebus_imitator-1.0], squirrel monkey; [saiBol1] and owl monkey; [Anan_2.0]) within the Cebidae family were obtained from NCBI and analyzed for their *Alu* content using RepeatMasker [13] (RepeatMasker-Open-4.0). Full-length elements were parsed from the RepeatMasker output using a custom python script. Full-length elements were defined as being 267 bp or longer and starting no more than 4 bp from the 5′ start of the *Alu* consensus sequence [13] (RepeatMasker-Open-4.0). Full-length elements, with 600 bp of 5′ and 3′ flanking sequence, for each genome were then compared against the human genome using BLAT [14]. Lineage specificity compared to the human genome (*Homo sapiens*; GRCh38.p13) for each Cebidae genome was determined using a custom python script to determine if the appropriate *Alu* gap size was present between the query sequence and target human genome that would indicate the element was only in the query sequence. For each set of cebid *Alu* loci, the orthologous sequence was obtained from the three remaining platyrrhine genomes via BLAT, aligned with MUSCLE [15] and placed into an alignment file. The average *Alu* insertion is ~300 bp, which includes the 3′ A-rich tail. With the 1200 bp total flanking sequence, an ideal alignment would be a total of 1500 bp. To analyze the sequences and their alignments for *Alu* elements accurately, only alignments containing 1500–1600 bp were analyzed. Custom python scripts were used to analyze the alignment data. Briefly, each sequence from the four-way sequence alignment was scored based on the presence or absence of an *Alu* by aligning an *Alu*S consensus sequence to the aligned sequence from each of the four genomes and each alignment was parsed into a category based on the presence of an *Alu* in an orthologous position in another genome (Table 1).

The alignments were completed using the following parameters (using BioPython’s PairwiseAligner): match_score=1.3; mismatch_score=0; target_open_gap_score=-1.0; target_extend_gap_score=-1.0; target_left_open_gap_score=-2; target_left_extend_gap_score=0; target_right_open_gap_score=-1; target_right_extend_gap_score=0; target_internal_open_gap_score=-5; target_internal_extend_gap_score=-3; query_open_gap_score=-5; query_extend_gap_score= -3; query_left_open_gap_score=-1; query_left_extend_gap_score=-1; query_right_open_gap_score=-2; query_right_extend_gap_score=0; query_internal_open_gap_score=-5; query_internal_extend_gap_score=-3.

### 2.2. Sequence Alignment Inspection

Each alignment output for a given category (Table 1) was sorted in Excel by genome coordinates to identify any overlapping information, i.e., the same insertion ascertained from multiple genome sets. These were eliminated to obtain a dataset of only unique candidate insertions for each category of shared insertions. Then, multi-locus four-way sequence alignments for each category were opened in BioEdit (version 7.2.5.) [16] and visually inspected for accuracy. Shared *Alu* insertions were validated based on having the same genomic position, target site duplications (TSDs) and general sequence congruence. These were retained for possible polymerase chain reaction (PCR) analyses. Those determined not to be shared insertions post-inspection were classified either as a near parallel insertion (NP) or Other: (N’s in the sequence; truncated sequence of undetermined status or having portions of the *Alu* in all four aligned genomes).

### 2.3. Oligonucleotide Primers for PCR

Following visual inspection of four-way sequence alignments, at least four candidate loci from each of ten phylogenetically informative categories were selected for oligonucleotide primer design. For the datasets in which an *Alu* insertion appeared to be shared in three genomes and absent from the fourth, oligonucleotides for PCR were attempted for all those that passed inspection. Forward and reverse oligonucleotide primers for PCR were designed using Primer3 (v.0.4.0) [17] and checked with the alignment in BioEdit [16] to ensure minimal mismatches and analyzed using NCBI Primer Blast [18] for primer specificity and predicted PCR amplicon length (Supplementary File S1). The oligonucleotide primers were obtained from Sigma Aldrich (Woodlands, TX, USA).

### 2.4. DNA Samples

DNA samples are described in Supplementary File S1. The DNA panel contained sixteen platyrrhine species representing all three families, Cebidae, Atilidae and Pitheciidae, as well as three outgroups. This DNA panel was used to screen elements for shared *Alu* insertions.

### 2.5. PCR Amplification

PCR amplifications were performed in 25 µL reactions containing 25 µg of template DNA, 200 nM of each primer, 1.5 mM MgCl_2_, 10× PCR buffer (1×: 50 mM KCl; 10 mM Tris-HCl, pH 8.4), 0.2 mM dNTPs, and 1 unit of *Taq* DNA polymerase. The PCR cycling protocol is as follows: 94 °C for 1 min, 32 cycles of denaturation at 94 °C for 30 s, 30 s at the appropriate annealing temperature (typically 57 °C), extension at 72 °C for 30 s, followed by a final 72 °C extension step for 2 min. Gel electrophoresis was performed on a 2% agarose gel containing 0.2 µg/mL ethidium bromide for 60 min at 180 V. UV fluorescence was used to visualize the DNA fragments using a BioRad ChemiDoc XRS imaging system (Hercules, CA, USA). Gel images were exported for publication as *.tiff files and uploaded to PowerPoint for annotation.

## 3. Results

### 3.1. Shared Alu Insertions

**Figure 1:**
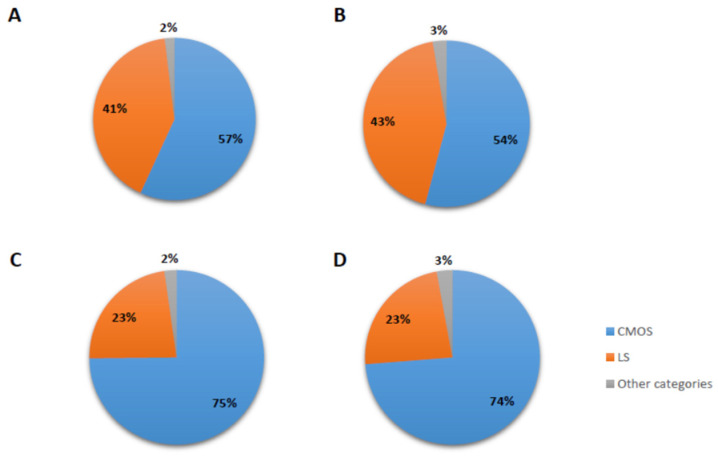
Distribution and genome comparison of shared and lineage-specific *Alu* insertions. Alignments were categorized into pre-defined groups (See Materials and Methods) and then broadly characterized into three groups: LS (lineage-specific) in orange indicates the percentage of the *Alu* insertions that were not found in orthologous position in any other genome. CMOS in blue indicates the percentage of the elements from the BLAT analysis that were shared by all four Cebidae genomes. (**A**) marmoset (**B**) squirrel monkey (**C**) owl monkey (**D**) capuchin monkey. The small gray pie slices are the percentages of *Alu* insertions within any of the other ten pre-defined shared categories.

The input values for full-length *Alu* sequences from marmoset; [caljac3], owl monkey; [Anan_2.0], squirrel monkey; [saiBol1] and capuchin monkey; [Cebus_imitator-1.0] were 61,513, 77,564, 32,145, and 58,952, respectively. These elements were obtained by extracting full-length *Alu* elements from each of the four Cebidae genomes in this study and only keeping elements that were lineage-specific when compared to the human genome. After orthologous sequence extraction and subsequent alignment, 51,320, 65,119, 28,614 and 49,093 aligned elements remained in the marmoset, owl monkey, squirrel monkey and capuchin monkey, respectively. After imposing a 1500 to 1600 bp sequence limit upon the alignments, 35,680, 39,349, 19,919, and 31,479 alignments remained for elements obtained from the marmoset, owl monkey, squirrel monkey and capuchin monkey, respectively. The *Alu* elements extracted from each of the four cebid genomes and subsequent alignment analysis indicated that the majority of the elements were either shared among all four genomes or were lineage-specific to the ascertained genera (Figure 1).

Shared *Alu* insertions represented by the gray pie slices in Figure 1 were parsed into sequence alignment files (Supplementary File S2) and visually inspected for each of the other ten pre-defined categories. These results are summarized in Table 2.

**Figure 2:**
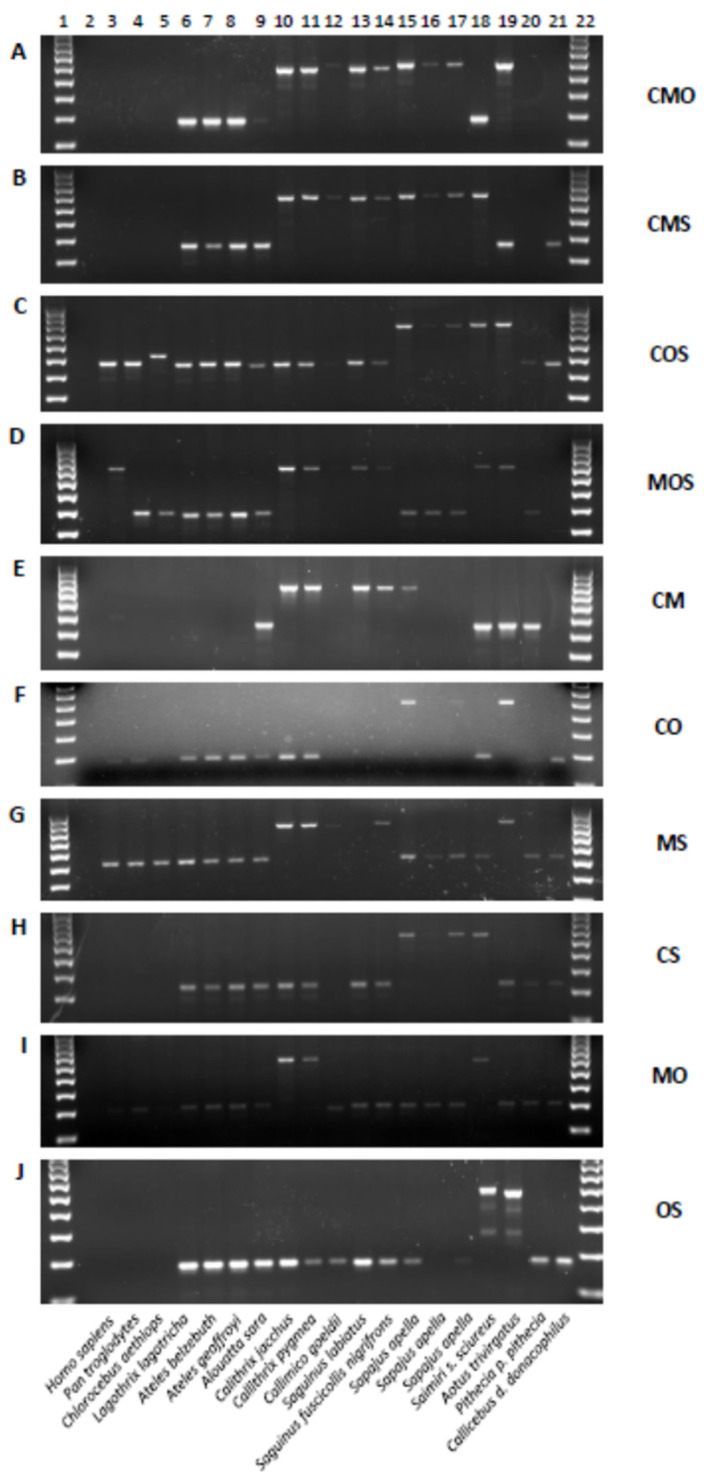
PCR analysis of phylogenetically informative *Alu* element categories. (**A**) CMO_blat_2; (**B**) CMS_blat_1; (**C**) COS_blat_4; (**D**) MOS_blat_2; (**E**) CM_blat_2; (**F**) CO_blat_4; (**G**) MS_blat_4; (**H**) CS_blat_2; (**I**) MO _blat_2; (**J**) OS_blat_4. Lanes: 1—100 bp ladder; 2—TLE (negative control); 3—Human (HeLa); 4—Chimpanzee; 5—African green monkey; 6—Wooly monkey; 7—White-bellied spider monkey; 8—Black-handed spider monkey; 9—Bolivian red howler monkey; 10—Common marmoset; 11—Pygmy marmoset; 12—Goeldi’s marmoset; 13—Red-chested mustached tamarin; 14—Geoffroys saddle-back tamarin; 15–17—Capuchin monkey; 18—Squirrel monkey; 19—Owl monkey; 20—Northern white-faced saki; 21—Bolivian gray titi; 22—100 bp ladder. Scientific names of the primates are indicated below the gel images. Letters on the right side of the gel image correspond with those found in Table 1. Loci names, PCR primers and DNA samples are available in Supplementary File S1.

These computational analyses and subsequent manual inspection were complemented by locus specific PCR assays. PCR results confirmed the presence of *Alu* insertions in all ten phylogenetically informative categories (Figure 2).

### 3.2. Alu Subfamily Distribution

**Figure 3:**
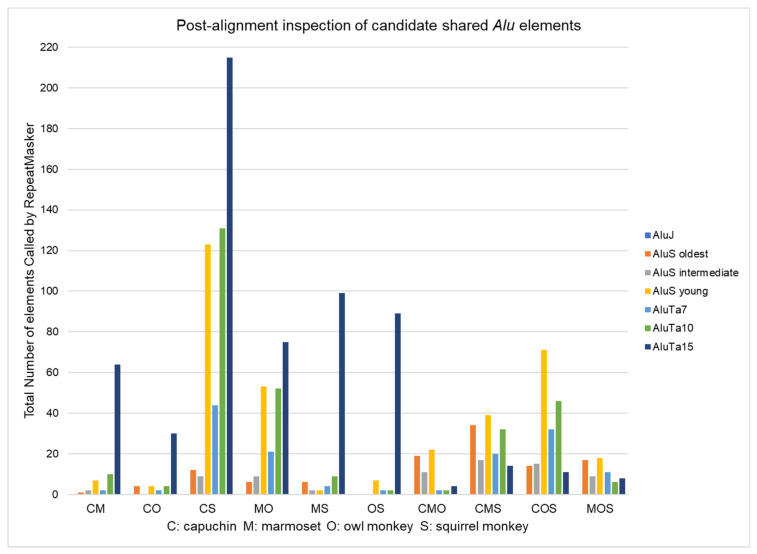
*Alu* subfamily distribution of post-alignment shared *Alu* insertions for each of ten pre-defined categories. CS: shared by capuchin and squirrel monkey to the exclusion of marmoset and owl monkey has the most elements. *Alu*Ta15 and derived younger subfamilies dominate recent *Alu* expansion in the two-genome categories, while four-way sequence alignments in which an *Alu* insertion appeared to be shared in three genomes and absent from the fourth are more broadly represented by both older *Alu*S and younger *Alu*Ta subfamilies.

*Alu* insertions that were determined to be shared following post-alignment inspection were analyzed using RepeatMasker for *Alu* subfamily distribution (Supplementary File S1; worksheet “RM Shared”; Table S1; Figure 3). The results for the oldest *Alu* subfamily, *Alu*J, include subfamilies (Jb, Jo, Jr and Jr4). RepeatMasker did not identify any *Alu*J elements in any of the ten pre-defined shared categories. The older *Alu*S subfamilies (Sp, Sq, Sq2, Sq10, Sx, Sx1, Sx3, Sx4, Sz, Sz6), the intermediate *Alu*S subfamilies (Sg, Sg4, Sg7), and the youngest *Alu*S subfamily branch, Sc (Sc, Sc5 and Sc8) are shown along with the platyrrhine specific *Alu*Ta subfamilies Ta7, Ta10 and Ta15.

The number of candidate *Alu* insertions listed as ‘unique calls’ in Table 2 is further distributed into post-alignment inspection groups in Table 3 to delineate reasons why a ‘unique call’ failed post-alignment visual inspection.

**Figure 4:**
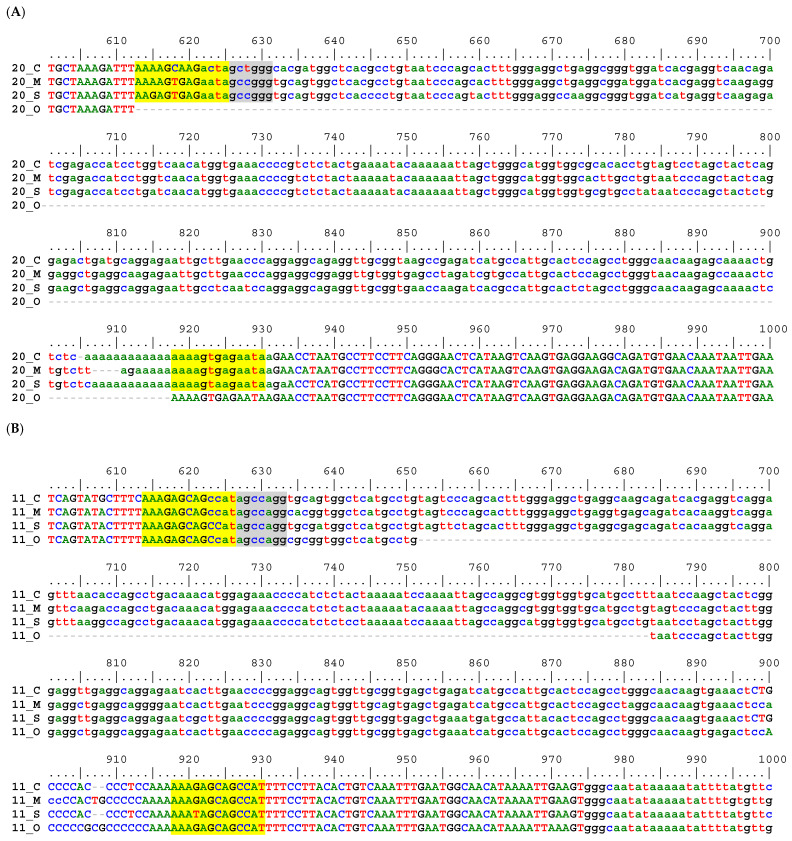

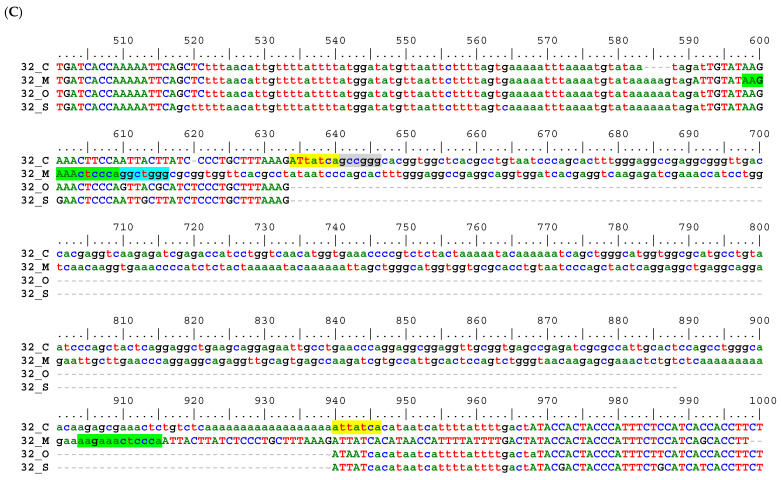
Genome alignments showing examples of predicted shared *Alu* insertions and post-alignment inspection results. (**A**) Locus CMS-20 the target *Alu* (start position in gray highlight) is shared by capuchin, marmoset, and squirrel monkey to the exclusion of owl monkey (precise pre-integration site) with matching flanking sequence and TDSs in yellow highlight. (**B**) Locus CMS-11 was predicted to be the same as in A, however upon inspection of the alignment the owl monkey sequence displays portions of the target *Alu* sequence, both after the start position (gray highlight) and before the TSDs (yellow highlight), while lacking the insertion sequence only between positions 652–784. Thus, the target *Alu* is actually shared by all four CMOS. (**C**) Locus CM-32 was predicted to be shared by capuchin and marmoset, to the exclusion of owl monkey and squirrel monkey. The target *Alu* from the capuchin genome [Cebus imitator_1.0] starts at position 641 (gray highlight), is flanked by TSDs in yellow highlight, while owl monkey and squirrel monkey display precise pre-integration sites. However, the marmoset sequence has a different *Alu* insertion, a near parallel insertion (NP) starting at position 610 (aqua highlight) with TSDs in bright green highlight.

Sequence alignment examples for each of these three post-alignment inspection groups are shown in Figure 4. An example of a validated shared insertion is shown for Locus CMS-20 (Figure 4A). An example of a failed inspection due to the target *Alu* insertion being present in all four aligned genomes is shown for Locus CMS-11 (Figure 4B), and an example of an NP event is shown for Locus CM-32 (Figure 4C).

### 3.3. Sequence Alignment-Based Phylogeny

Four-way sequence alignments in which an *Alu* insertion appeared to be shared in three genomes and absent from the fourth were comprehensively investigated to potentially determine the phylogenetic placement of owl monkey (genus *Aotus*). These four categories were: (1) COS, shared by capuchin, owl monkey, and squirrel monkey to the exclusion of marmoset; (2) CMS, shared by capuchin, marmoset, and squirrel monkey to the exclusion of owl monkey; (3) MOS (absent in capuchin); or (4) CMO (absent in squirrel monkey). Following visual inspection of alignments, only about 10–20% were retained as actually being shared by three species and absent from the fourth genome (Table 2). Many of the failed candidates had N’s in the sequence, had truncated sequences for one or more of the genomes, or had portions of the *Alu* in all four species of the alignments. These cases were excluded from further experiments. PCR analyses were conducted on all remaining candidates that PCR primers could be designed for, and at least half from each category (*n* = 98 total) with emphasis on CMS (absent from owl monkey, suggesting that *Aotus* is basal within Cebidae) or COS (absent from marmoset, suggesting that Callitrichinae—marmoset and tamarin—are basal within Cebidae), as these two categories were most likely to be true based on the number of alignment-producing candidates (Table 2).

**Figure 5:**
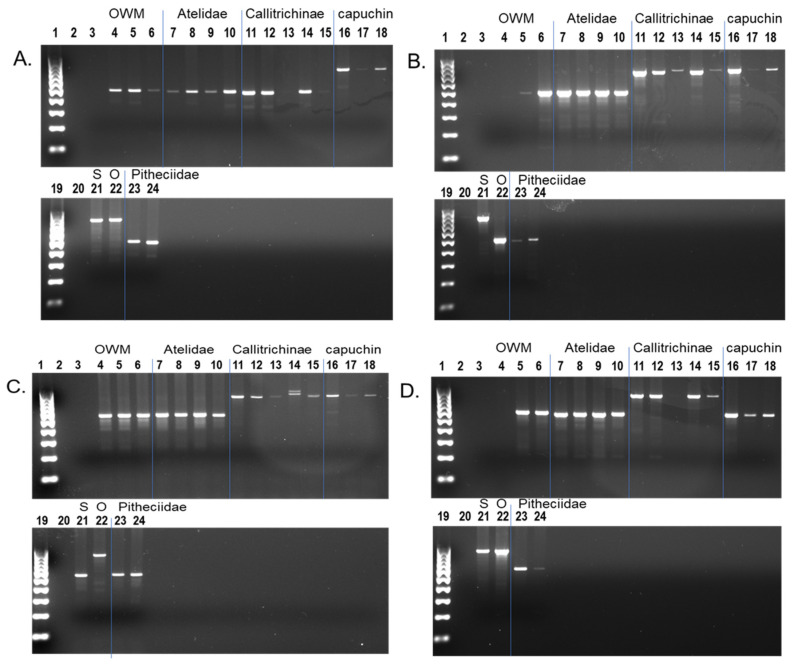
PCR analyses of *Alu* elements shared in three of four cebid genera and also absent in Atelidae and Pitheciidae. Lanes: 1—100 bp DNA ladder; 2—blank; 3—TLE (negative control); 4—Human (HeLa); 5—*Pan troglodytes* (common chimpanzee); 6—*Chlorocebus aethiops* (African green monkey); 7—*Lagothrix lagotricha* (woolly monkey); 8—*Ateles belzebuth* (white bellied spider monkey); 9—*Ateles geoffroyi* (black-handed spider monkey); 10—*Alouatta sara* (Bolivian red howler monkey; 11—*Callithrix jacchus* (common marmoset); 12—*Callithrix pygmea* (Pygmy marmoset); 13—*Callimico goeldii* (Goeldi’s marmoset); 14—*Saguinus labiatus* (red-chested mustached tamarin); 15—*Saguinus fuscicollis nigrifrons* (Geoffroys saddle-back tamarin); 16–18 *Sapajus apella* (tufted capuchin); 19—100 bp DNA ladder; 20—blank; 21—*Saimiri s. sciureus* (common squirrel monkey); 22—*Aotus trivirgatus* (Three striped owl monkey); 23—*Pithecia p. pithecia* (Northern white-faced saki); 24—*Callicebus d. donacophilus* (Bolivian gray titi monkey). (**A**) COS #51, *Alu* is present capuchin, owl monkey and squirrel monkey (~790 bp DNA fragment lanes 16–18, 21–22) and absent in marmosets and tamarins (~465 bp DNA fragment lanes 11–15). This *Alu*Sc supports callitrichines (marmosets and tamarins) as basal within Cebidae. (**B**) CMS #64, *Alu* is present in capuchin, marmosets, tamarins and squirrel monkey (~800 bp DNA fragment lanes 11–18, 21) and absent in owl monkey (~496 bp DNA fragment lane 22). This *Alu*Sc supports owl monkey (*Aotus*) as basal within Cebidae. (**C**) CMO #29, *Alu* is present in capuchin, marmosets, tamarins and owl monkey (~860 bp DNA fragment lanes 11–18, 22) and absent in squirrel monkey (~525 bp DNA fragment lane 21). This *Alu*Ta7 supports squirrel monkey as basal within Cebidae. (**D**) MOS #5, *Alu* is present in marmosets, tamarins, owl monkey and squirrel monkey (~880 bp DNA fragment lanes 11–15, 21–22) and absent in capuchins (~564 bp DNA fragment lanes 16–18). This *Alu*Ta7 supports capuchin as basal within Cebidae.

These PCR results are summarized in Table 4. The COS category has the highest numbers with 63 *Alu* elements passing alignment inspection out of 286 candidates and 16 that were confirmed by PCR out of 40 tested by gel electrophoresis (Figure 5A). These results indicate that marmoset is most basal in Cebidae out of the four genomes in this study. However, the CMS category placing *Aotus* basal to the other three genomes is almost as likely with 14 confirmed by PCR out of 32 (Figure 5B), with a validation rate of 44% compared to 40% for the COS group. The other two categories, CMO (Figure 5C) and MOS (Figure 5D), meaning that squirrel monkey or capuchin is basal, respectively, have far less support and are considered very unlikely based on phylogenetic studies. However, the fact that all four categories have PCR confirmed shared *Alu* insertions suggests that the radiation of these genera was very rapid, causing extensive incomplete lineage sorting (ILS) of *Alu* elements that had not yet reached fixation. PCR results that did not confirm the predicted relationship most often showed the *Alu* present in all platyrrhine DNA samples, indicative of sequence quality anomalies, or had poor amplification.

Subfamilies *Alu*Sc and *Alu*Ta7 were both active simultaneously with the divergence of platyrrhines and catarrhines [1] about 20 mya. The Ta-lineage is unique to platyrrhines. These data are consistent with ILS of *Alu* insertions that occurred after the Cebidae divergence from Atelidae and Pitheciidae but immediately prior to the rapid speciation of cebid taxa. These *Alu* elements remained unfixed within Cebidae and became randomly assorted for presence or absence in subsequent emerging species. Still, both COS and CMS seem equally likely, thus indicating that the radiation of *Aotus* and the Callitrichinae (leading to marmoset and tamarins) occurred at nearly the same time. Rapid radiation about 20 mya resulted in extensive ILS of *Alu* elements that were not fixed in the various populations at the time of speciation and became randomly assorted for presence or absence in these cebid lineages. Shared *Alu* insertions in this study were often identified as *Alu*Sc or *Alu*Ta7 subfamilies which were mobilizing at the time of radiation. These insertions are roughly 20 my old, and while some sequence decay was observed in the alignments, the *Alu* start and stop positions and TSDs could usually be identified. RepeatMasker identified shared insertions from multiple genomes as being from the same *Alu* subfamily or derived from the same subfamily (i.e., both Ta10 or both Ta15 derived) the majority of the time (Supplementary File S1; worksheet “RM Shared”; Table S1). The consensus sequences for these different *Alu* subfamilies often vary by only 1–2 bp and could result from 20 my of random genome decay, while still being the same original insertion. However, in a few cases, RepeatMasker identified some members as being from a different subfamily lineage (i.e., one Ta10 derived and the other Ta15 or Sc derived but look shared by alignment to the same location). Although it is possible that these represent random genomic decay of a shared insertion, they could also indicate incidents of “Precise Parallel Insertions” in which a different *Alu* element integrated into the exact same genomic position in different cebid genomes. These are much more difficult to delineate compared to near parallel insertions (NP), particularly after potentially 20 my of evolution and further impair phylogenetic interpretation.

## 4. Discussion

The analysis of *Alu* elements ascertained from the marmoset, squirrel monkey, capuchin monkey and owl monkey genomes based on orthologous sequence alignments provide strong evidence of ILS. PCR confirmation of the presence of *Alu* insertions in all phylogenetically informative categories indicates that ILS is widespread among cebid taxa. ILS is likely a product of the rapid speciation that occurred within platyrrhines during which time a large number of *Alu* insertions remained polymorphic within the emerging taxa and became randomly distributed among the four lineages studied here. ILS resulting from a large effective population size has also been proposed as having a predominant role in cebid phylogeny, perhaps more so than short interval speciation events [9]. The effective population size of the common ancestor of *Aotus* and callitrichines (marmosets and tamarins) is reportedly one of the highest among primates [9]. Both scenarios generate high levels of genetic polymorphisms and ILS at speciation resulting in incongruent phylogenetic trees [8,9,19,20]. The PCR analyses confirmed that each phylogenetically informative group is represented by having shared *Alu* insertions that were predicted in the alignment data sets. In addition, the sequence data present in the assembled genomes provided higher levels of orthology with which to unambiguously assign *Alu* insertions to a phylogenetically informative group. This is in contrast with the minimal orthology provided by SRA data alone [11]. The alignment data also provides information on truncated *Alu* elements and near parallel insertions. Near parallel insertions can obscure a phylogenetic analysis if not carefully considered [21]. While shown to be rare in primates, precise parallel insertions are also possible within this alignment data set based on the *Alu* subfamily analysis. For example, the subfamily of a shared element between capuchin monkey and owl monkey to the exclusion of squirrel monkey and marmoset can be determined with this expanded subfamily data set. If the subfamilies of both *Alu* insertions from the capuchin monkey and owl monkey in the alignment are the same subfamily or closely related, it is more likely that this is truly a shared element rather than a precise parallel insertion. Alternatively, if the subfamilies from the capuchin monkey and owl monkey differ, it is probable that a precise parallel insertion took place. A complex species radiation coupled with an abundance of *Alu* proliferation impairs complete resolution of Cebidae phylogeny using this strategy.

However, this alignment approach provided some evidence for resolving Cebidae evolutionary relationships. Previous studies based on both morphology and retrotransposable element insertion presence have indicated a close relationship between the capuchin monkey and the squirrel monkey [22,23,24,25]. The CS category contained the highest number of shared *Alu* insertions compared to any other combination of taxa based on sequence alignments. Further, *Aotus* and the Callithrichinae (marmosets and tamarins) are most likely basal to either squirrel monkey or capuchin monkeys, when considered separately.

Rapid diversification and large ancestral population size among cebid taxa have made determining the exact phylogeny a difficult task that has required innovative methods to be applied, which have yet to give confident results. A platyrrhine specific SINE element called Platy-1 is reported to have little activity in *Aotus*, virtually no current mobilization in squirrel monkey and capuchins [26], while marmoset exhibits extensive expansion of Platy-1 elements [27]. This could mean that a branch including *Aotus*, that later led to squirrel monkey and capuchins, diverged first, followed by the Callitrichinae branch that led to marmosets and tamarins and that the Platy-1 expansion took place after this split. This phylogeny was supported by Osterholz et al. (2009) [5], using *Alu* elements. It could also mean that they emerged nearly simultaneously and the Platy-1 radiation in marmoset was simply delayed.

Future work on generating high-quality assemblies should be a priority, as highlighted by gaps and Ns (where the sequence quality was not high enough to assign a nucleotide) seen in this study. This effort may include generating high quality assemblies using a variety of sequencing methods, which may include long sequencing technologies such as PacBio [28] or NanoPore [29], in conjunction with Illumina sequencing [30] and Hi-C sequencing [31]. A combination of these methods have been used to reproduce fully assembled genomes or higher quality than previous versions, such as the gray mouse lemur [32]. However, it seems unlikely that improved future genome assemblies alone would substantially impact the conclusions of this study given such widespread ILS.

## 5. Conclusions

This study represents the most extensive use of *Alu* genetic systems to date within the Cebidae family of platyrrhine primates. The close phylogenetic relationship between squirrel monkey (genus *Saimiri*) and capuchins (*Cebus* and *Sapajus*) is well supported while the placement of owl monkey (*Aotus*) with respect to the marmosets and tamarins (Callithrichinae) remains unresolved. Presence of *Alu* insertions in all phylogenetically informative *Alu*-shared categories is evidence of extensive ILS during the complex radiation among cebid taxa. Identification of multiple near parallel insertions as well as possible precise parallel insertions within this dataset implies that *Alu* proliferation further impairs phylogenetic resolution using this strategy.

## Figures and Tables

**Table 1:** **Possible alignment output categories.** The first column shows all the possible combinations. ‘C’, ‘M’, ‘O’ and ‘S’ indicate that an *Alu* insertion is present in the capuchin monkey, marmoset, owl monkey, or squirrel monkey genome, respectively. An ‘x’ in a row indicates the taxa in that category that would share an *Alu* insertion, while the exclusion of an *Alu* candidate from an organism is indicated by ‘n/a’ in a gray box in that row. Table adapted from [11] with permission from Elsevier.

	Capuchin Monkey	Marmoset	Owl Monkey	Squirrel Monkey
CMOS	x	x	x	x
CMO	x	x	x	n/a
COS	x	n/a	x	x
CMS	x	x	n/a	x
MOS	n/a	x	x	x
CM	x	x	n/a	n/a
CO	x	n/a	x	n/a
CS	x	n/a	n/a	x
MO	n/a	x	x	n/a
MS	n/a	x	n/a	x
OS	n/a	n/a	x	x
C	x	n/a	n/a	n/a
M	n/a	x	n/a	n/a
O	n/a	n/a	x	n/a
S	n/a	n/a	n/a	x

**Table 2:** **Distribution of shared *Alu* insertions for each of ten Cebidae categories.** First column definitions: Genome ascertained: C—capuchin; M—marmoset; O—owl monkey; S—squirrel monkey. Set # is the genome the *Alu* was ascertained from, each with the number of candidate elements per set in the next row. These are combined (a.) for total candidates in each of the ten shared categories (bold). If the same locus appeared in multiple genome sets, these duplicates (b.) were removed to obtain only unique calls for each category (a–b). Then, each alignment was visually inspected for accuracy of the shared insertion prediction. The number that passed inspection for each category are shown in the row ‘post-alignment inspection’ and as a percentage of the number of unique calls.

Genome Ascertained	C	M	C	O	C	S	M	O	M	S	O	S	C	M	O	C	M	S	C	O	S	M	O	S
Set #	1	2	3	4	5	6	7	8	9	10	11	12	13	14	15	16	17	18	19	20	21	22	23	24
Candidates by set	95	75	39	53	342	175	122	184	154	90	146	50	97	101	147	169	127	85	168	220	79	85	123	47
Shared category	**CM**		**CO**		**CS**		**MO**		**MS**		**OS**		**CMO**			**CMS**			**COS**			**MOS**		
a. Total Candidates	170		92		517		306		244		196		345			381			467			255		
b. In multiple sets	38		19		106		83		42		31		153			143			181			96		
Unique calls (a–b)	132		73		411		223		202		165		192			238			286			159		
Post-align Inspection	43		22		267		108		61		50		20			52			63			23		
% retained	33%		30%		65%		48%		30%		30%		10%			22%			22%			14%		

**Table 3:** **Distribution of Post-alignment inspection results for each of ten Cebidae categories.** The values for rows “Unique calls” and “Post-align shared” are the same as in Table 2. Post-align NP is the number of unique calls that upon visual inspection were determined to be near parallel insertions (NP) rather than shared. Post-align other* means that there were N’s in the sequence; the sequence was truncated with undetermined status, or portions of the *Alu* were present in all four aligned genomes. These numbers are totaled and shown as a percentage of the total number of unique calls in the two right-hand columns.

Shared Category	CM	CO	CS	MO	MS	OS	CMO	CMS	COS	MOS	Total	%
Unique calls	132	73	411	223	202	165	192	238	286	159	**2081**	
Post-align shared	43	22	267	108	61	50	20	52	63	23	**709**	**34.1%**
Post-align NP	45	22	44	77	99	81	3	5	2	4	**382**	**18.4%**
Post-align other*	44	29	100	38	42	34	169	181	221	132	**990**	**47.6%**

**Table 4:** Summary of PCR results for Post-aligned shared in three of four genomes.

Shared Category	CMO	CMS	COS	MOS	All
Post-align shared	20	52	63	23	**158**
Analyzed by PCR	10	32	40	16	**98**
PCR confirmed	4	14	16	3	**37**
% confirmed by PCR	4/10 (40%)	14/32 (44%)	16/40 (40%)	3/16 (19%)	**37/98 (38%)**

## Data Availability

The Supplementary data files are available on the online version of this paper and through the Batzer Lab website under publications, https://biosci-batzerlab.biology.lsu.edu/ (accessed on 19 October 2022).

## Supplementary Materials

The following supporting information can be downloaded at: https://www.mdpi.com/article/10.3390/life12101655/s1, Supplementary File S1 is an Excel file containing the worksheets for PCR primers, DNA samples, the RepeatMasker output for the shared *Alu* elements for each category and Table S1. Supplementary File S2 is a *.zip file containing the multi-locus four-way sequence alignments used in this study. Table S1. RepeatMasker summary for Post-align shared *Alu* subfamily determination.
